# Targeting the Cysteine Redox Proteome in Parkinson’s Disease: The Role of Glutathione Precursors and Beyond

**DOI:** 10.3390/antiox12071373

**Published:** 2023-06-30

**Authors:** Marcos A. Martinez-Banaclocha

**Affiliations:** Department of Pathology, Lluis Alcanyis Hospital, Xátiva, 46800 Valencia, Spain; martinez_marben@gva.es

**Keywords:** aging, cysteine, glutathione, n-acetyl-cysteine, proteome, Parkinson, redox, rejuvenation, senescence

## Abstract

Encouraging recent data on the molecular pathways underlying aging have identified variants and expansions of genes associated with DNA replication and repair, telomere and stem cell maintenance, regulation of the redox microenvironment, and intercellular communication. In addition, cell rejuvenation requires silencing some transcription factors and the activation of pluripotency, indicating that hidden molecular networks must integrate and synchronize all these cellular mechanisms. Therefore, in addition to gene sequence expansions and variations associated with senescence, the optimization of transcriptional regulation and protein crosstalk is essential. The protein cysteinome is crucial in cellular regulation and plays unexpected roles in the aging of complex organisms, which show cumulative somatic mutations, telomere attrition, epigenetic modifications, and oxidative dysregulation, culminating in cellular senescence. The cysteine thiol groups are highly redox-active, allowing high functional versatility as structural disulfides, redox-active disulfides, active-site nucleophiles, proton donors, and metal ligands to participate in multiple regulatory sites in proteins. Also, antioxidant systems control diverse cellular functions, including the transcription machinery, which partially depends on the catalytically active cysteines that can reduce disulfide bonds in numerous target proteins, driving their biological integration. Since we have previously proposed a fundamental role of cysteine-mediated redox deregulation in neurodegeneration, we suggest that cellular rejuvenation of the cysteine redox proteome using GSH precursors, like N-acetyl-cysteine, is an underestimated multitarget therapeutic approach that would be particularly beneficial in Parkinson’s disease.

## 1. Introduction

Despite the significant progress in understanding the pathophysiology of Parkinson’s disease (PD), its prevention and treatment remain without significant therapeutic advancement. PD is a prevalent neurodegenerative illness resulting in progressive motor impairment and cognitive dysfunction [1]. Most PD cases are sporadic, and only a low percentage is related to mutations in a few genes, causing familial PD [2,3,4,5]. As in other prevalent neurodegenerative disorders, aging is the principal risk factor for developing this condition [6]. Toxic compounds and genetic mutations likely play a central function in PD development and progression [2,3,4]. However, the pathophysiology of PD onset and progression is not fully understood.

PD patients develop a progressive loss of dopaminergic neurons in the substantia nigra of the brain, exhibiting neuronal Lewy bodies formed by protein aggregates whose principal component is α-synuclein [7,8]. The α-synuclein accumulation seems to start as microaggregates at the presynaptic level, likely interfering with neurotransmitter vesicle trafficking and release in dopaminergic cells [9,10]. However, Lewy bodies also appear in asymptomatic aged people [11], indicating they are assembled in aggresome-related processes to rescue neurons from protein misfolding [12].

The molecular intricacy of PD denotes that multiple biochemical pathways underlie the clinical manifestations and progression of the disease [2,3,4,5], expressing the involvement of oxidative damage, reduced antioxidant ability, and mitochondrial dysfunctions related to cellular senescence in dopaminergic neurons [13,14,15].

To determine the intricate connections among the diversity of biochemical pathways intervening in the pathophysiology of PD, we have proposed the disturbance of the cysteine redox proteome [16] as the essential underlying pathogenic hub that likely explains this complexity, opening the possibility of new therapeutic options [17]. Although most neurodegenerative diseases, including PD, show differences in symptomatology and evolution, which seem typical of each disorder, they share proteomic deregulation associated with redox disturbances and the accumulation of specific proteins that finally end in neuronal death. Additionally, glutathione (GSH) precursors have proven beneficial effects in diabetes, hypertension, obesity, and other chronic comorbidities that contribute to neurodegeneration, and recent clinical trials have shown a significant benefit of N-acetyl-cysteine (NAC) in PD, probably related to the upregulation of the cysteinome. Since the combination of many different molecular pathways plays a critical role in the mechanisms of cell rejuvenation and regeneration, the present article has focused on adding and complementing scientific evidence supporting the potential beneficial effect of GSH precursors against the deregulation of the redox proteome in PD.

## 2. The Cysteine Redox Proteome Hub (Cysteinome and Cysteinet)

**Figure 1:**
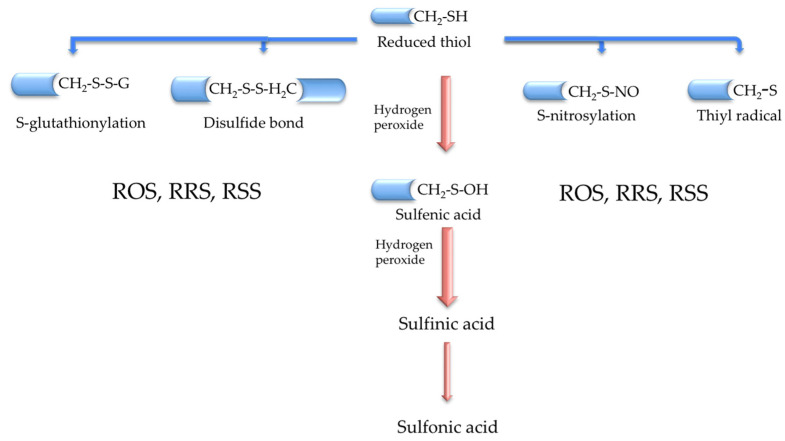
Schematic representation of the main thiol redox reactions in proteins.

The redox proteome encloses all proteins that can suffer redox changes relying on the functional status of some amino acids such as Trp, Tyr, Arg, Cys, Met, and seleno-cysteine [16,17]. The cysteine redox proteome (cysteinome) is an essential piece of the redox proteome [17,18,19], and its dysfunction has been defined as cysteinet deregulation [17]. Cysteinet includes all sensitive cysteine-containing proteins (SCCPs) interconnected with reactive oxygen (ROS), nitrogen (RNS), and sulfur (RSS) species, regulating cellular survival, regeneration, and death [17]. Therefore, each SCCP works as a molecular sensor synchronizing, in real-time, most metabolic routes via the reversible redox shift in protein function [17]. Indeed, cysteinet is crucial in brain development [20], and its disruption in neurodegeneration is under the control of dysfunctional SCCPs and reactive species [20,21,22,23,24]. Although the amount of cysteine residues in proteins is low, they are vital to keep the synthesis, arrangement, and diversity of proteins and small peptides, like GSH. Sensitive cysteines in proteins operate in manifold redox reactions (Figure 1), including the catalytic action in enzymatic complexes of the mitochondrial respiratory chain, the structural organization of the cytoskeletal proteins, the folding and transport of proteins across membranes, and the regulation of DNA synthesis and expression, including translation, histone modifications, and mRNA splicing [19,25].

Protein homeostasis or proteostasis is crucial for supporting the optimal dynamic cellular proteome, comprising the synchronization of protein synthesis, post-translational modification and trafficking to the adequate subcellular compartment, the assemblage in protein complexes, and the final degradation into amino acids [26,27]. The post-translational modification of proteins (PTMPs) enriches the complexity and diversity of proteins in particular tissues specifying the functionality of most biochemical routes in the cell. PTMPs comprise, among others, the redox modifications of some specific amino acids in proteins. However, cysteine is the most versatile motif because its thiol group (-SH) is deprotonated under physiological conditions, allowing redox molecular switches. Proteins can exhibit reversible or irreversible redox modifications in response to different oxidant compounds and pH conditions [26,28,29]. Also, the cellular antioxidant machinery (thioredoxins, glutaredoxins, peroxiredoxins) utilizes reversible transitions in their catalytic cysteine redox states to restore redox homeostasis [29,30]. Accordingly, the exquisite management of cysteine redox changes on proteins participates in the accurate protein folding, functionality, and secretion via palmitoylation, prenylation, s-nitrosylation, and s-glutathionylation (Figure 1) [29,30]. Moreover, radical transfer protects against oxidative damage in proteins, concerning a chain of electron transfer among redox residues, such as cysteine or methionine, to counteract the oxidative impact at the initial point [31]. This mechanism operates at the catalytic places of some enzymes and catalytic complexes like the mitochondrial respiratory chain [32].

Therefore, cellular-sensitive cysteine residues in proteins maintain the thiol homeostasis through the exquisite dialogue with extracellular cysteine, which is located principally as disulfide cystine because of the relative oxidizing extracellular conditions (Figure 1). This thiol pool complements the principal intracellular cysteine-containing small peptide GSH, participating in an intricate network of enzymatic reactions in all cellular compartments [33]. Glycine, cysteine, and glutamate integrate GSH in two enzymatic steps. Cysteine is combined with glutamate through glutamate-cysteine ligase activity to produce γ-glutamylcysteine that reacts with glycine through glutathione synthase [33]. Generally, GSH can reduce disulfide links in proteins under physiological conditions and produce glutathione disulfide (GSSG), reduced back using NADPH as the electron donor via glutathione reductase. Likewise, GSH is a cofactor in multiple antioxidant enzymes, like glutathione reductases, glutathione peroxidases, and glutathione s-transferases [33].

Recent data on the molecular pathways behind rejuvenation in a metazoan at the post-reproductive stage recognized variants and expansions of genes related to DNA replication and repair, telomere and stem cell maintenance, redox microenvironmental regulation, and intercellular communication [34]. Moreover, life cycle reversal showed the silencing of the transcription factors polycomb repressive complex 2 (PRC2) targets and the triggering of the pluripotency targets [34], indicating that hidden networks integrate multiple molecular mechanisms to maintain and synchronize the optimal inter-regulation among the genetic repertoire, the transcriptional ability, and protein functional communication. Although cysteine represents only 2% of the total amino acids in proteins, it is significantly conserved through evolution, participating in complex organism regulation [19] and the aging process [35,36]. Indeed, aging shows gradual and cumulative somatic mutations, telomere attrition, epigenetic modifications, and oxidative deregulation, ending in the loss of clonal diversity across the lifespan, which could be partially due to cysteinet deregulation.

We propose the optimization of the redox proteome through rejuvenation of the cysteinome is vital to counteract the detrimental effects of senescence in associated degenerative diseases, particularly in PD. In this regard, we present evidence about the potential therapeutic role of GSH precursors and other unrelated molecules to restore the cysteinet deregulation in this disorder.

## 3. The Cysteinet Deregulation in Aging

Aging and degenerative disorders show dysregulation in the redox homeostasis of proteins, dysfunctional mitochondria, biomolecular oxidative injury, and aggregation of misfolded proteins into distinct subcellular organelles [17,26,28]. The oxidative damage of macromolecules relies on reactive species overproduction accompanied by a decline in cysteine/cystine, total GSH content, and low GSH/GSSG proportion. These findings imply an age-associated modification of redox homeostasis toward oxidative conditions, influencing numerous regulatory cellular pathways that depend on the structural and functional integrity of SCCPs [37,38,39,40,41]. Accordingly, senescence boosts irreversible damage to proteins modifying normal cellular functions and homeostasis [17]. For example, aging shows a harmful shift in redox-regulated glycolytic enzymes and regulatory proteins controlling the energetic metabolism in muscular cells [38]. Also, physiological brain aging displays an accumulation of characteristic proteins like Aβ-amyloid and tau aggregation [39] and reduced mitochondrial function with a decrease in mitochondrial gene expression in humans with cognitive decline [40]. Hence, age-prone oxidative damage of SCCPs may play a fundamental role in senescence and neurodegenerative disorders [17].

As previously seen, proteostasis entangles protein homeostasis across all cellular organelles, including the cytoplasm, cytoskeleton, mitochondria, Golgi apparatus, and endoplasmic reticulum [26,28,42]. Cysteine residues give the appropriate disulfide bonds to support the 3D configuration and stability of synthesized proteins in the endoplasmic reticulum [43]. It seems that the endoplasmic reticulum environment evolves more reduced while the cytosol is more oxidizing in stressing conditions and aging [26,28,42]. Therefore, the redox environment in the endoplasmic reticulum ensures the accurate folding of proteins, which is exquisitely maintained by a complex network of oxidases and disulfide isomerases [26,28,42].

Degradation of damaged proteins depends on the initial ubiquitination and binding to the E1 ubiquitin-activating enzyme by a thiol ester linkage [44], followed by transferring the started ubiquitin to the E2 ubiquitin-conjugating enzyme through a functional cysteine in the E2 protein [44,45]. Ubiquitin E3 ligases also hold sensitive cysteines that switch their function. Furthermore, the redox-regulated E3 ligase adaptor Kelch-like ECH-associated protein 1 (Keap1) has many susceptible cysteines interacting with Nrf2 [46,47]. Nrf2 is an essential mediator of the cellular antioxidant response integrating signals from aggregated proteins to coordinate a fine transcriptional function [48,49]. Thus, the ubiquitin E3 ligase-Keap1 complex acts as a redox detector guiding Nrf2 ubiquitination and degradation. Additionally, Nrf2 regulates crucial proteins linked to the GSH metabolism in the brain, including cystine/glutamate transport, γ-glutamate-cysteine synthetase (γ-GS), glutamate-cysteine ligase, glutathione reductase (GR), and glutathione peroxidase (GPX) [49]. Also, the protein folding in the endoplasmic reticulum relies on sulfhydryl oxidases as disulfide donors, such as protein disulfide isomerase (PDI) and endoplasmic oxidoreductin 1 (Ero1). PDI catalyzes disulfide bonds between redox-reactive cysteines to assist protein folding. For example, Cys663 oxidation in the kinase activation loop of the endoplasmic reticulum protein IRE-110 inhibits the extended protein reaction stimulating the p38 mitogen-activated protein kinase (MAPK) antioxidant pathway [48].

Cysteinet deregulation in aging and neurodegenerative disorders needs detailed investigation. Hydrogen peroxide, nitric oxide, hydrogen sulfide, NADP/NADPH, free cysteine/cystine, and GSH/GSSG feedback this cellular redox network and the impairment of its balance impact the protein folding, leading to redox deregulation affecting protein proteostasis [17,50].

## 4. Cysteinet Deregulation in PD

PD pathophysiology involves many proteins from the autophagy-lysosome and α-synuclein aggregation pathways, indicating that a failure in proteostasis likely plays an essential role in this disorder. Indeed, cysteine residues are more oxidized in dopaminergic cells of the substantia nigra than in other regions in physiological conditions and PD, reinforcing the importance of cysteinet regulation in this specific area of the brain in PD [17,23,24,51,52,53,54,55,56].

### 4.1. Extracellular Vesicles

**Figure 2:**
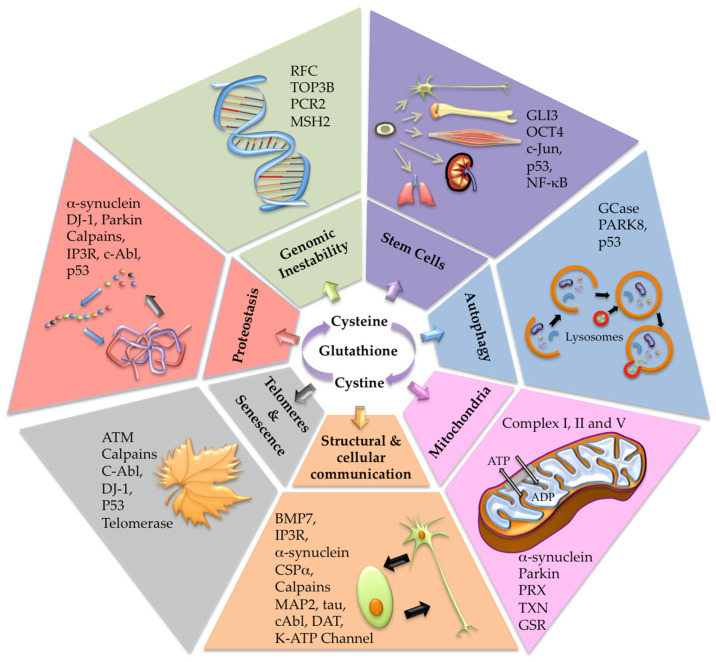
Schematic overview of the cysteinome participation in senescence and PD. Cysteine residues are conserved at functional sites within proteins, performing diverse roles in metal binding, catalysis, redox chemistry, and intercellular communication. Sensitive cysteine-containing proteins (SCCPs) function in structural and cellular communication, efficient telomere and proteostasis maintenance, genomic stability, stem cell rejuvenation, autophagy, and mitochondrial bioenergetics. Abbreviations: ATM, ataxia-telangiectasia mutated; BMP7, bone morphogenetic protein 7; c-Abl, cellular-abelson tyrosine kinase; CSPα, cysteine string protein a; DJ-1, protein deglycase DJ-1; ETC, electron transport chain; GCase, glucocerebrosidase; GLI3, GLI family zinc finger 3; GSR, glutathione-disulfide reductase; IP3R, IP3 receptor; MSH2, MutS homolog 2; NF-κB, nuclear factor kappa light chain enhancer of activated B cells; OCT4, octamer-binding transcription factor 4; Parkin, E3 ubiquitin ligase; PRC2, polycomb repressive complex 2; PRX, peroxiredoxin; RFC, replication factor C; TOP3B, DNA Topoisomerase III beta; TPP1, tripeptidyl-peptidase 1; TXN, thioredoxin.

Proteostasis implicates extracellular vesicle protein transport among cells through exosomes and microvesicles [57]. Cells under oxidative stress maintain the redox balance by releasing cysteine-oxidized proteins via exosomes as a defensive tool against oxidative injury [57,58,59]. Specifically, exosome liberations under oxidative situations rely on the redox control of the inositol 1,4,5-trisphosphate receptor (IP3R), initiating Ca^2+^ discharge from the ER into the cytoplasm [57]. Reciprocally, microvesicle and exosome release depend on Ca^2+^-influx through transmembrane channels containing sensitive cysteines [60]. IP3R contains numerous functional cysteine residues that may suffer modifications by ROS belonging to the ER and the mitochondria [61]. This receptor regulates cytoplasmic Ca^2+^ levels via the modulation of sensitive cysteines to adapt the active structural form of the protein complex [62]. IP3R and ryanodine receptors (RYRs) are the principal neuronal Ca^2+^ channels regulated by the redox modification of sensitive-cysteine residues (Figure 2). Specifically, IP3R has cysteine residues in the catalytic domain as well as three specific cysteine residues (Cys56, Cyc849, and Cys2214) that are involved in the palmitoylation of the receptor [61,62]. Palmitoylation involves the addition of the fatty acid palmitate to cysteine residues via a thioester bond facilitating the membrane association of IP3R. On the other hand, RyR1 contains at least seven redox-sensitive cysteines (Cys1040, Cys1303, Cys2436, Cys2565, Cys2606, Cys2611, and Cys3635), with Cys3635 being the s-nitrosylation site [60].

Moreover, Ca^2+^-dependent stimulation of calpains (nonlysosomal Ca^2+^-dependent cysteine proteases) enables microvesicle building under the redox modification of sensitive cysteines [63]. It is important to remember that calpains significantly participate in vital neuronal functions, including the remodeling of cytoskeletal/membrane attachments, diverse signal transduction pathways, and apoptosis [64]. Calpain immunoreactivity is highly expressed in diverse specific areas in the brain of PD patients but not in other neurodegenerative disorders or controls [65]. Therefore, deregulated calpain systems following cysteinet disruption and loss of Ca^2+^ homeostasis may participate in PD pathophysiology (Figure 2).

### 4.2. DnaJ Homolog C (DNAJC)

The α-synuclein aggregates travel via axons to the closest neuronal cells or are cleared by the ubiquitin–proteasome machinery [66,67]. DNAJC is a subtype of heat shock protein (HSP) whose mutations are related to PD and other parkinsonian illnesses [68,69]. DNAJC5 encodes cysteine string protein α (CSPα) mainly localized in presynaptic vesicles to guarantee the proper folding of synaptic proteins [68,69] and interacting with α-synuclein to sustain the soluble NSF attachment protein receptor (SNARE) complex assemblage, which merges vesicles with the target membrane [70,71,72]. CSPα contains a string-domain-containing 13–15 heavily palmitoylated cysteines that have linkage purposes (Figure 2). The redox changes in this region result in complex aggregates of CSPα, demonstrating that the palmitoylation of specific cysteine residues is required for the aggregation of CSPα [73].

Further, high neuronal activation raises the discharge of tau protein via exosomes, boosting its transfer to adjacent cells [74,75]. Also, the dissemination of tau via exosomes is high in PD, indicating that the spreading of proteins among neuronal cells participates in the disorder’s advancement [76].

### 4.3. Glucocerebrosidase

Around 10% of PD patients show mutations in the lysosomal enzyme glucocerebrosidase (GCase), but only some GCase-mutated carriers develop the illness [77,78]. Additionally, some patients of nonfamiliar PD without GCase mutations display reduced enzyme levels, indicating the probable interactions between autophagy disruptions and α-synuclein cumulation in PD [77,78]. Moreover, wild-type GCase function may alleviate PD phenotypes in animal models. On the other hand, GCase activation in pluripotent-stem-cell-derived dopaminergic cells from sporadic PD improves the lysosomal activity, reducing the accumulation of oxidized dopamine, glucosylceramide, and α-synuclein [79]. Similar results showed patients bearing mutations in the genes encoding the GCase, LRRK2, DJ-1 (PARK7), or parkin displaying low GCase activity [79] (Figure 2).

GCase can be imported into the mitochondria facilitating the keeping of mitochondrial complex I integrity and function [80]. Furthermore, GCase disease-associated mutations may damage complex I resilience, interfering with the mitochondrial quality control machinery [80]. These mitochondrial actions of GCase may contribute to complex I impairment and the consequent defective energy metabolism that causes dopaminergic cell death.

GCase has many functional cysteines in various GCase regions. Only one cysteine (Cys1465) is in the GTPase domain, four (Cys2024, Cys2025, Cys2101, and Cys2114) are in the kinase domain [81], and three participate in the GCase activity, being modulated by GSH [82]. Moreover, highly oxidized dopamine can control GCase function through cysteine residues in its functional domain [83], implying the redox shift of reactive cysteines [82,83].

### 4.4. Leucine-Rich Repeat Kinase 2

The kinase enzyme leucine-rich repeat kinase 2 (LRRK2), called PARK8, is found in the cytoplasm and the mitochondrial outer membrane participating in cell autophagy (Figure 2). PARK8 contains two crucial cysteines for redox sensing in the activation loop of the protein [84]. Variants and mutations of this gene increase the likelihood of PD development [85]. Studies in idiopathic PD show that ROS activates PARK8 in dopaminergic neurons, suggesting that oxidative modification of this protein elicits the phosphorylation of its Rab10 substrate, resulting finally in neuronal death [86]. Therefore, redox deregulation of the LRRK2 pathway has a role in PD pathophysiology [86]. Interestingly, 2-hydroxybutyrate levels in the CSF from idiopathic and LRRK2-related PD patients are low [87]. This compound belongs to the threonine or methionine metabolism through homocysteine and cystathionine, producing cysteine, which may reestablish GSH levels and synthesis [87].

### 4.5. Cellular-Abelson Tyrosine Kinase (c-Abl)

c-Abl protects cells against oxidative damage by sensing ROS [32,88] through pivotal sensitive cysteines, which intervene in the building of disulfide bonds that inhibit c-Abl activation, demonstrating that the redox modifications of reactive cysteine residues in c-Abl are essential for its function [89]. Recent investigations establish that the c-Abl pathway participates in PD development via the phosphorylation of α-synuclein, promoting its aggregation and interfering with its discharge via the ubiquitin–proteasome machinery [88]. Also, parkin phosphorylation via c-Abl may inhibit the parkin ligase activity, interfering with the proteasome process and increasing dopaminergic degeneration [88]. Scientific evidence shows that c-Abl confers proapoptotic reactions to DNA injury via pathways that rely on p53 and its homolog p73 [90] (Figure 2). Accordingly, c-Abl inhibitors are potential substances in the treatment of PD [88,91].

### 4.6. Parkin

E3 ubiquitin ligase (parkin) is a component of the RBR (RING-between-RING) ubiquitin ligase family that intervenes in the proteasome and lysosome ubiquitination machinery (Figure 2). Some parkin domains (RING0, RING1, RING2, and the in-between-RING (IBR)) are cysteine-rich, which are needed to attach to eight Zn^2+^ ions [92]. In addition to Cys431 in the catalytic region of parkin, Cys268 and Cys323 recognize defective mitochondrial membrane proteins that intervene in mitophagy [93,94]. Parkin functional cysteines were found to be s-nitrosylated in the brains of patients with parkinsonism interfering with its protective action [95]. S-nitrosylation modulates parkin’s susceptibility to oxidative dopamine damage by modifying Cys268 and Cys323 reactive residues on the protein [95], and the s-nitrosylation of parkin may improve neuronal survival, reducing apoptosis [96,97]. Also, parkin mutations are associated with mitochondrial disruptions contributing to neuronal death in PD [92,98,99].

### 4.7. Dopamine Transporter (DAT)

DAT is a trans-membrane protein implicated in dopamine reuptake at presynaptic terminals to promote its release. Consequently, DAT regulates dopamine signals for dopamine homeostasis [100] (Figure 2). DAT has eight reactive cysteines in the hydrophilic loops of the protein set on both sides of the cellular membrane. Some of these cysteine residues are essential for maintaining the fine 3D structure of the dopamine translocation transporter [101], whereas other cysteine residues function in s-palmitoylation through thioester bonds [102].

### 4.8. Protein Deglycase DJ-1

DJ-1 protein (encoded by PARK7 gen) is a cysteine protease found to be mutated in autosomal-recessive PD patients which also participates in sporadic PD cases [103]. Oxidative damage inactivates DJ-1 in patients with sporadic PD and AD. DJ-1 has three sensitive cysteine residues (Cys46, Cys53, and Cys106) susceptible to s-nitrosylation. However, the oxidative change of Cys106 specifically regulates the protein function, disturbing its antioxidant activity in dopaminergic neurons [104]. Furthermore, the s-nitrosylation of Cys106 in DJ-1 hampers PTEN transnitrosylation, raising the phosphatase activity and lowering neuronal survival [105] (Figure 2).

### 4.9. ATP-Sensitive Potassium Channel (K-ATP Channel)

K-ATP channels have four potassium canals composing the pore and four sulfonylurea receptors (SUR1 or SUR2). Hydrogen sulfide controls dopamine release via the redox shift of two reactive cysteine residues (Cys6 and Cys26) found in the N-terminal region of the regulatory SUR1 domain of the complex [106,107] (Figure 2). Therefore, the oxidative modifications of K-ATP channels interfere with dopamine excitability, being involved in the vulnerability of neuronal disturbances in PD [108].

### 4.10. Antioxidant Enzymatic System

In complement to the GSH enzymatic regulation, functional cysteines control other essential antioxidant enzymes (Figure 2). Peroxiredoxins (PRXs) are antioxidant enzymes holding sensitive cysteines in the catalytic center that reduces peroxides generated in the metabolism with the cooperation of the thioredoxin system [109]. Indeed, oxidative modifications at Cys51 and Cys172 among peroxiredoxin-2 proteins can induce intermolecular disulfide bonds reversed by the thioredoxin system. However, further oxidative changes in Cys51 and Cys172 can block peroxiredoxin-2 antioxidant action against hydrogen peroxide, facilitating dopaminergic neuronal death [109]. Interestingly, PD brains show more s-nitrosylated peroxiredoxin-2 levels than controls [109]. Peculiarly, thioredoxin (TXN) and GSH systems control diverse cellular functions, including the transcription machinery [17]. TXN proteins contain two catalytically active cysteines that can reduce disulfide bonds in numerous target proteins, controlling their biological function. Glutathione-disulfide reductase (GSR) is a cysteine redox enzyme of the GSH system, contributing to maintaining and regulating the cellular redox state [17].

### 4.11. Mitochondrial Respiratory Chain and Oxidative Phosphorylation

**Figure 3:**
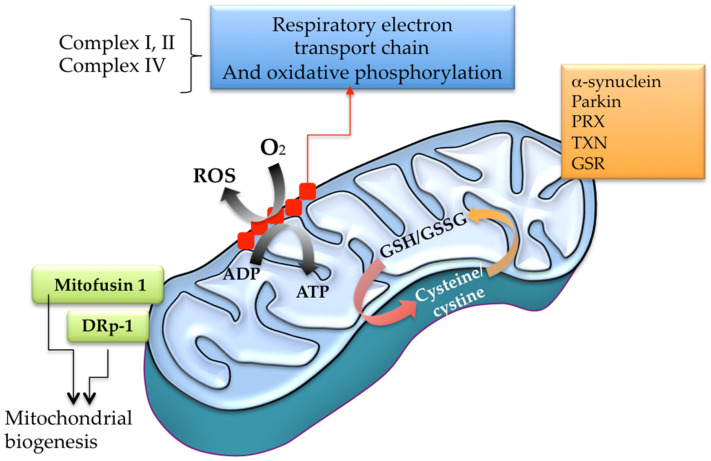
Representation of some critical mitochondrial SCCPs that participate in cysteinet deregulation in PD.

Many intermediary metabolic enzymes are redox-regulated through cysteine thiol group modifications (Figure 3). In the mitochondria, some irreversible changes result in the impairment of specific respiratory chain complexes resulting in further ROS over-production [110] (Figure 3). ROS, RNS, and RSS can modify mitochondrial complexes into the oxidative phosphorylation machinery. Complex I has various sensitive cysteines (Cys39, Cys137, Cys531, and Cys704), and complex II also has sensitive Cys90, Cys267, Cys476, and Cys537. The irreversible oxidative damage in some reactive cysteines on those complexes may interfere with the electron flux, increasing ROS generation and decreasing ATP levels (Figure 2 and Figure 3). Also, cysteine disulfide bond formation between mitochondrial complex V subunits can significantly reduce ATP synthesis. Indeed, the disulfide link between Cys294 and Cys103 in complex V inhibits its activity, and the oxidative shift of Cys294 in the α-subunit via s-glutathionylation or s-nitrosylation can alter the nucleotide-binding process, lowering ATP synthesis [110].

### 4.12. Microtubule-Associated Protein Tau and MAP2

Tau–tau links depend on sensitive cysteines to facilitate the link between the microtubule-binding domains of the proteins [111]. Nonetheless, Cys322 can be redox-modified, allowing the pathological assembly of tau into neurofibrillary tangles to spread under oxidative stress [112,113]. Vitamin B_12_ can interact with tau protein, blocking these cysteine residues and hampering tau aggregation [114]. Therefore, in addition to the imbalance between protein kinases and phosphatases leading to tau fibrillation, aging and other toxic or metabolic factors can shift cysteine oxidation, raising tau fibrillation in neuronal cells (Figure 2). Moreover, sensitive cysteine oxidation in tau and MAP2 can be induced via peroxynitrite and H_2_O_2_, altering their assembly with microtubules and enabling their pathological accumulation [115] (Figure 2). MAP2 is highly vulnerable to calpain activity, degrading it in response to Ca^2+^ and destabilizing the microtubules in the neuronal bodies and dendrites [116] (Figure 2).

Tau protein catalyzes its intramolecular and intermolecular auto-acetylation via sensitive cysteine residues in the microtubule binding area [117]. In healthy neurons, 99% of tau protein has no acetyl-transferase function because tau proteins are linked to microtubules. Hence, tau auto-acetylation is an event that promotes tau aggregation [117], and compounds like methylthioninium prevent tau filaments precursors via oxidative modification of their reactive cysteines [118]. Thus, molecules that attach to tau-reactive cysteines may control neurofibrillary tangle-related brain disturbance [111,119], suggesting that redox modulation of this essential protein may prevent its accumulation in PD.

### 4.13. α-Synuclein and the Ubiquitin–Proteasome System

The actual effects of α-synuclein misfolding, aggregation, and toxicity are unidentified. The α-synuclein participates in synapsis function through mitochondrial homeostasis, supporting neurotransmitter release and reuptake and controlling vesicle motion and storage (Figure 2). Thus, α-synuclein misfolding and agglomeration interfere with normal mitochondrial bioenergetics, raising the oxidative impairment of dopaminergic neurons.

Reactive species can induce α-synuclein assembly, causing durable crosslinking dimers [120]. Since α-synuclein does not possess cysteine or tryptophan, oxidative injury can affect tyrosine and methionine residues, inducing protein fibrillation and aggregation [120]. In addition to ROS overproduction in dopaminergic cells, α-synuclein’s mutations may boost its assemblage by altering its secondary structure contributing to protein misfolding [121]. Moreover, oxidized α-synuclein is not easily removed by the ubiquitin–proteasome system, which is disturbed by the cysteinet dysfunction itself when GSH levels are low since this system employs the successive action of diverse SCCPs [12,122] (Figure 2). The E1 enzyme starts an energy-rich thioester bond that involves the C-terminal glycine of the ubiquitin protein and the sensitive cysteine of the active center of the E1 enzyme, permitting the identification and degradation of the ubiquitin-labeled protein [123]. Additionally, reactive cysteines modulate the E3 ubiquitin ligase (parkin) activity, controlling mitochondrial proteins through the proteasome machinery [93] (Figure 2).

The proteasome triggers transcription regulators, such as the nuclear factor-kappa B (NF-κB) signaling pathway. Interestingly, oxidative stress activates the NF-κB pathway and suppresses autophagy and autophagy-dependent apoptosis, enabling the assembly and outspreading of α-synuclein [124]. Indeed, α-synuclein accumulation in mitochondria produces deficiencies in cellular respiration [125] that may interfere with the mitochondrial fusion machinery via SCCPs [20,24], such as mitofusin-1 (Mfn1), mitofusin-2 (Mfn2), and optic atrophy type 1 (Opa1), allowing mitochondrial fragmentation [126] (Figure 2).

### 4.14. Pluripotency and Neural Stem Cells

Subventricular zone adult neural stem cells (NSCs) gain a pluripotent state after the octamer-binding transcription factor 4 (OCT4) overexpression permitting the differentiation into midbrain dopaminergic (DA) neurons [127]. OCT4-induced pluripotent DA neuron transplantation enhanced the behavioral motor deficiencies in PD’s animal models. OCT4 contains nine cysteines, four (Cys185, Cys198, Cys221, and Cys252) placed in the N-terminal region. The two DNA binding domains each contain two cysteines (Figure 2). One final cysteine is present in the linker region between both DNA binding domains, regulating its DNA binding under redox regulation [128]. Interestingly, GSH prevents the oxidation of OCT4 cysteines and their subsequent degradation, allowing OCT4 to bind DNA [128].

Transcription factor DNA binding regulation via redox cysteine modification also operates for other transcription factors, like p53, activator protein 1 (AP-1), and NF-κB [128]. Increased concentrations of p53 were observed in PD brains and animal models, supporting the association between p53 activation and dopaminergic degeneration in this disorder [129,130]. p53 activation in response to neurodegenerative stress is closely related to dopaminergic neuronal death linked with mitochondrial dysfunction, ROS overproduction, autophagy deregulation, and impaired protein assembly, integrating the cellular stress response [130]. The p53 DNA binding domain includes several redox-sensitive cysteines, where oxidation diminishes the DNA binding of p53 [128]. Moreover, site-directed mutagenesis of these cysteines resulted in a total failure of the p53 DNA binding process [128,131] (Figure 2).

The c-Jun N-terminal kinases are activated by neurotoxins and environmental stress, potentially contributing to PD pathophysiology [132]. Indeed, cFos-cJun heterodimers cannot bind to DNA after the oxidation of one single bit of cysteine residue necessary for DNA binding [132] (Figure 2).

NF-κB is also involved in PD [133]. The α-synuclein aggregation triggers NF-κB activation in neuronal cells, inducing apoptosis through several mechanisms. Additionally, misfolded α-synuclein liberated from deteriorated neurons starts some signaling pathways in glial cells culminating in NF-κB activation and overproduction of pro-inflammatory cytokines, exacerbating neurodegeneration [133]. Furthermore, NF-κB activation is essential for adequate mitochondrial function, and its inhibition contributes to dopaminergic neuron loss through different mechanisms [133]. NF-κB activity depends on a single redox-sensitive cysteine (Cys62) required for DNA binding [133] (Figure 2).

The transcription factor GLI3 is a member of the canonical Hedgehog (HH) signaling route, an integral modulator of tissue patterning and development [134]. Intriguingly, GLI3 shows that all copies are transcriptionally active during the life cycle reversal of some metazoans [34]. The Cys609 of GLI3 directly coordinates a zinc ion, mediating conformational changes that inhibit GLI3 proteolysis and maintaining its activation [134]. Cys609 mutations in GLI3 inhibit its post-transcriptional repressor (GLI3R), also inducing a weak transcriptional activation that may competitively interfere with the GLI2 and GLI1 function [135] (Figure 2).

### 4.15. Cysteinet Deregulation and Genomic Instability

Topoisomerase III (Top3) is the only topoisomerase that can change the topology of DNA and RNA in mammals, playing an essential role in neurodevelopment and mental dysfunction in humans [136]. Top3β mutations are related to schizophrenia, autism, epilepsy, and cognitive dysfunction. Indeed, Top3β knockout mice show behavioral phenotypes similar to some psychiatric disorders and cognitive impairments, displaying defects in hippocampal neurogenesis and synaptic plasticity. Moreover, Top3β knockout mutant mice show damaged global neuronal activity-dependent transcription in response to stress, including multiple neuronal processes [137]. Therefore, Top3β activity is vital for physiological brain function, and the disturbance of neuronal activity-dependent transcription may permit Top3β mutations to cause cognitive impairment and psychiatric disorders [138]. Cysteine residues are conserved in the extreme C-terminus of the Top3β, at nonrandom positions, to coordinate zinc ions. These zinc-finger-containing domains typically serve as interactors to bind DNA, RNA, and other proteins, contributing to their function [138] (Figure 2).

Transcription factors also regulate cell-type-specific gene programs to support neuronal individuality (Figure 2). PRC2 is a protein complex essential for the epigenetic regulation of gene expression that consists of four core subunits, including the complex with histone methyltransferase activity [139]. The histone methyltransferase (HMTase) activity of the carboxy-terminal SET domain of the PRC2 requires two adjacent Zn^2+^ cysteine-rich complexes (including a cysteine-rich CXC domain in the enzymatic subunit) regulating the catalytic function [140].

PRC2 permits accurate differentiation during the maturation of the CNS [141] but is also persistently expressed in differentiated neurons facilitating the neuronal identity despite the changing conditions. Furthermore, PRC2 maintains neuronal-specific gene repression, allowing chromatin condensation [141]. In this context, PRC2’s adequate function maintains the identity and activity of midbrain dopaminergic neurons. Consequently, the loss of PRC2 activity causes phenotypes that mimic the essential characteristics of PD symptoms without compromising neuronal survival [141].

### 4.16. Ataxia-Telangiectasia Mutated (ATM) and Apoptosis

Ataxia-telangiectasia mutated (ATM) is a serine/threonine protein kinase triggered by DNA damage. ATM is the principal regulator against double-strand breaks (DSBs) through ATM dimers’ dissociation into active monomers [142]. ATM is activated by Cys2991 oxidation on the disulfide-cross-linked dimer, contributing significantly to increased ATM activity in DSBs and oxidative stressing conditions [142] (Figure 2). This enzyme phosphorylates specific proteins (p53, CHK2, BRCA1, NBS1, and H2AX) that activate cell cycle arrest, DNA restoration, or apoptosis. Some studies indicated that neurotoxin 1-methyl-4-phenyl pyridinium (MPP^+^)-induced DNA damage initiates the ATM response in vitro. However, the selective inhibition of ATM is protective against MPP^+^-induced apoptosis, supporting the potential role of ATM in PD, which present DNA damage in brain samples [143]. Cellular stress induced by α-synuclein elicits DNA damage and activates the DNA damage response (DDR) [144]. The α-synuclein can cause the upregulation of three different markers of DNA damage (γH2AX, 53BP1, and ATM) in dopaminergic cells [144]. Oxidative species participate in DDR because exogenous and endogenous antioxidants inhibit DDR and rescue mitochondrial dysfunctions caused by α-synuclein. These results agree with previous evidence that α-synuclein oligomerization or oxidation can cause DNA breaks [143,144]. Moreover, recent findings show that ATM activation can prevent protein aggregation. Therefore, DDR activation via a-synuclein stress is a component of the cellular response against protein aggregation [143,144].

### 4.17. Bone Morphogenetic Protein 7 and TGF-β

Bone morphogenetic protein 7 (BMP7) prevents α-synuclein-induced loss of dopaminergic neurons, motor disabilities, and gliosis. Also, BMP5/7 therapy reduces α-synuclein accumulation [145]. BMP7 contributes to phosphorylate SMAD1 and SMAD5, which results in the transcription of multiple osteogenic genes [146]. The failure of BMP/SMAD signaling results in α-synuclein accumulation, suggesting that the therapeutic action of BMP5/7 involves the decrease of α-synuclein assemblage [147]. The BMP7 monomer contains the characteristic cysteine-knot domain of the transforming growth factor β (TGF-β) superfamily. This knot results from a fold induced by three intra-molecular disulfide bonds. A seventh cysteine residue (Cys103) neighbors the loop within the knot, allowing an intermolecular disulfide bond resulting in the mature active BMP7 heterodimer [147] (Figure 2).

### 4.18. Telomerase and Senescence

Telomeres are ribonucleoproteins constituted by repetitive DNA sequences and a combination of proteins indispensable for genomic stability. Oxidative damage induces telomere attrition, playing a potential role in neurodegenerative disorders, including PD [148]. Nevertheless, the contribution of telomere damage in the pathogenesis of neurodegenerative diseases requires more research [149].

Telomerase conserves the telomeres, catalyzing the elongation of the 3′ end of telomeric DNA [150], playing a principal role in senescence [151,152] by controlling the chromosomal stability and cellular viability [153,154]. Telomerase contains the catalytic human telomerase reverse transcriptase (hTERT) and the noncoding human telomerase RNA (hTR), providing the template for DNA elongation [155]. hTERT regulation involves transcriptional and post-transcriptional modifications [156,157]. Indeed, ROS overproduction promotes the hTERT export from the nucleus to the cytoplasm, participating in senescence and age-related neurodegenerative diseases [158,159]. hTERT includes four essential domains (TEN, TRBD, RT, and CTE), and hTR contains two central components needed for its function: the template/pseudoknot (t-PSK) and the CR4/5 domains. The telomerase catalytic center does not require cysteines [160]. Moreover, cysteine residues in the crossing-point (CP) and E motifs are not crucial for telomerase catalysis. However, though some sulfhydryl compounds show the covalent alkylation of the Cys528 of the TRBD and the Cys896 in the motif D of the RT domain [160], the building of mutants for these specific cysteines displayed physiological function and similar sensitivity as alkylating inhibitors, indicating that the sensitiveness of telomerase to sulfhydryl substances probably depends on modulating the enzymatic process targeting noncatalytic cysteine residues [160] (Figure 2).

## 5. Role of Glutathione Precursors in Parkinson’s Disease

**Figure 4:**
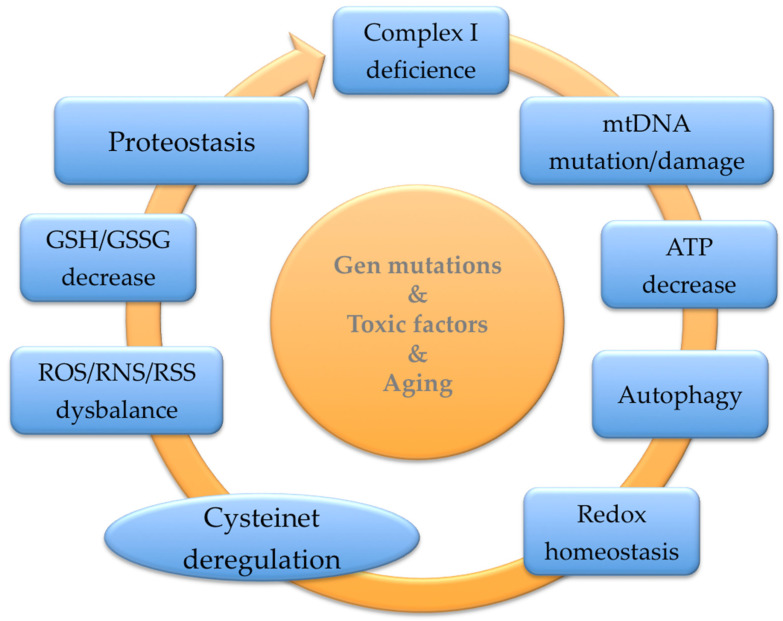
Summary of the main pathophysiological mechanisms that may be improved with GSH precursors.

Assuming the widespread distribution of functional cysteines in such a diverse kind of protein in cells, we have previously hypothesized that GSH precursors and compounds that modulate hydrogen sulfide metabolism would have a potentially beneficial therapeutic action on neurodegenerative diseases (Figure 4).

Redox modifications of sensitive cysteines sustain the thiol quality control of essential proteins, supporting cells against oxidative and xenobiotics damage (Figure 4). Cysteine is the rate-limiting substrate for the cellular tripeptide GSH (cysteine, glycine, and glutamic acid) synthesis. Cystine arrives in the cell via the xCT^(−)^ CSSG/L-glutamate antiporter (SLC7A11) system for maintaining GSH synthesis [33]. Despite low levels of H_2_S compared with other low-molecular-weight thiols, H_2_S is a powerful thiol working via the thioredoxin or NADPH systems to play its reducing effects [161]. Cells exposed to elevated H_2_S present higher GSH levels than the nontreated control, indicating an enhanced GSH recycling rate [162]. These data show that H_2_S maintains the cellular redox equilibrium defending cells from oxidative damage in GSH deficiency conditions [162].

GSH participates in the reduction of oxidative stress, the maintenance of redox equilibrium, the improvement of metabolic and organic pollutant detoxification, and the regulation of the immune system [17] (Figure 4). Clinical research indicates that nutritional interventions, including phytochemicals and foods, have pivotal effects on GSH’s proper balance. Moreover, individual genetic variability modifies the GSH levels influencing the global GSH status due to variability in enzymes implicated in its synthesis and renewal. Red blood cell GSH has a broad intra-individual divergence, though it is relatively long-lasting because of the variation the in genes controlling GSH concentrations [163].

### 5.1. Nutrients and GSH Status

#### 5.1.1. Amino Acids, Peptides, and Proteins

Many nutrients contribute to sustaining optimal GSH status. The intake of proteins affects the amino acid pool for synthesizing GSH. Indeed, supplementation with proteins with high cysteine content, such as whey protein, results in a dose-dependent boost in lymphocyte GSH concentrations [164,165]. Serine may positively impact GSH generation, increasing cysteine availability and reducing hyper-methylation, supporting the glycine metabolism for GSH synthesis.

GSH is easily oxidized, showing a short half-life in plasma (<3 min). Moreover, GSH does not efficiently cross cell membranes requiring elevated quantities to gain therapeutic concentrations [166]. Although digestive peptidases can degrade oral GSH showing no change in GSH concentrations or oxidative stress parameters [167], some clinical studies found substantial increases in the body’s GSH stores under different administrations. For example, using GSH monoesters improved its bioavailability [168].

#### 5.1.2. Vitamins

Some vitamins participate in the GSH status. Low doses of vitamin C can increase GSH levels in red blood cells [169]. Vitamin E administration may also increase GSH levels by reducing oxidative stress markers [170,171]. B2 vitamin is a necessary coenzyme for glutathione reductase, which restores the GSH from GSSG [172]. Moreover, the B5 vitamin may support GSH status via ATP generation [172]. Finally, alpha-lipoic acid can restore antioxidant capacity, including GSH regeneration [173,174].

#### 5.1.3. Flavonoids and Thiol-Rich Compounds

Many phytonutrients participate in the cellular antioxidant defenses that boost GSH metabolism, including foods containing all three thiol-rich compounds (GSH, NAC, and cysteine) [175]. NAC is concentrated in plants of the Allium species, especially in the onion. Therefore, eating a diet rich in asparagus, onion, cucumber, grapefruit, green and red pepper, strawberry, and tomato may easily contribute to redox homeostasis [175]. Xanthohumol is a flavonoid that contains an isoprenyl group that can regulate the Nrf2-ARE pathway in neuronal cells by covalently modifying cysteine residues in proteins. It can change the activity of glucose-6-phosphate dehydrogenase (G6PD), limiting the production of NADPH [176]. Xanthohumol can also hamper the generation of inflammatory intermediates like NO, IL-1β, and TNF-α, interfering with the activation of the NF-κB pathway [177].

#### 5.1.4. Michael Acceptor Molecules (MAMs)

In physiological circumstances, thiols and amines in small molecules or proteins can undergo a Michael addition reaction [178]. Diverse MAMs derived from plants have active electrophilic groups that target reactive residues on proteins with functional biological effects and low toxicity, providing some therapeutic options. Cysteine, homocysteine, GSH, and SCCPs are widespread reactive thiols that can interact with MAMs. MAMs can activate the Keap1-Nrf2-ARE pathway, playing antioxidant functions by covalently binding to sensitive cysteines. They also have anti-inflammatory functions through the inhibition of the NF-κB, a crucial sensitive cysteine protein modulated through redox reactions. These substances, used as dietary supplements, are effective for diverse oxidative stress-mediated inflammatory and developmental and degenerative diseases [179].

MAM-derived molecules such as andrographolide and lophirone B and C can modify the Keap1–Nrf2 pathway by alkylating specific reactive cysteines in Keap1, enhancing the cellular antioxidant ability and liver detoxification enzyme activities [180,181]. Moreover, they may enable the expression of NQO1, HO-1, UGT, SOD, GST, and EPH, improving cellular antioxidant and liver detoxification capacities [180]. Finally, Cardamonin can alter the functional cysteines of Keap1 through α, β-unsaturated carbonyl structure forming a covalent adduct, promoting Nrf2 nuclear translocation and triggering the expression of downstream genes of ARE [181]. Interestingly, Cardamonin can enter the blood–brain barrier, exerting a potential neuroprotective influence on ROS-associated neurodegenerative disorders ([181].

The α,β-unsaturated arrangements of MAMs can hamper the inhibitor of nuclear factor-kappa B kinase (IKK) by attaching to the reactive cysteines placed in the activation loop of IKKβ, inhibiting IκBα phosphorylation and therefore blocking the NF-κB route [180]. Moreover, physalin A can target Cys59, Cys179, Cys299, Cys370, Cys412, and Cys618 residues in the IKKβ loop resulting in anti-inflammatory effects [182]. Additionally, PKC has a cysteine-rich region and is the upstream enhancer of MAPK, the principal IKK activation signal [183]. Also, the α,β-unsaturated structure may interfere with the NF-κB pathway by linking to PKC cysteines [178]. Finally, Helenalin can interfere with the NF-κB route by binding to Cys38 of p65 [184].

### 5.2. CoQ10-Related Compounds

EPI-743 is a synthetic fat-soluble drug (para-benzoquinone) that is much more potent than CoQ10 or idebenone in protecting cells against oxidative damage in mitochondrial diseases [185]. Indeed, EPI-743 represents a new treatment for inherited mitochondrial respiratory chain disorders [186,187], and it is an antioxidant that crosses the blood–brain barrier, increasing glutathione synthesis [186,188]. Also, EPI-743 is used for some fatal inherited neurodegenerative disorders [189]. Compared to other antioxidant molecules, EPI-743 probably exerts a more intricate action, modulating multiple cell pathways, including Nrf2, a crucial cellular regulator against oxidative damage. Moreover, EPI-743 has shown improvement against oxidative stress markers that correlated with clinical improvement and a significant decrease in CNS glutamine/glutamate levels in PD patients [190]. These findings were associated with improvements in UPDRS scores that approached statistical significance [190].

### 5.3. N-Acetyl-Cysteine (NAC)

#### 5.3.1. NAC Bioavailability and Safety

**Figure 5:**
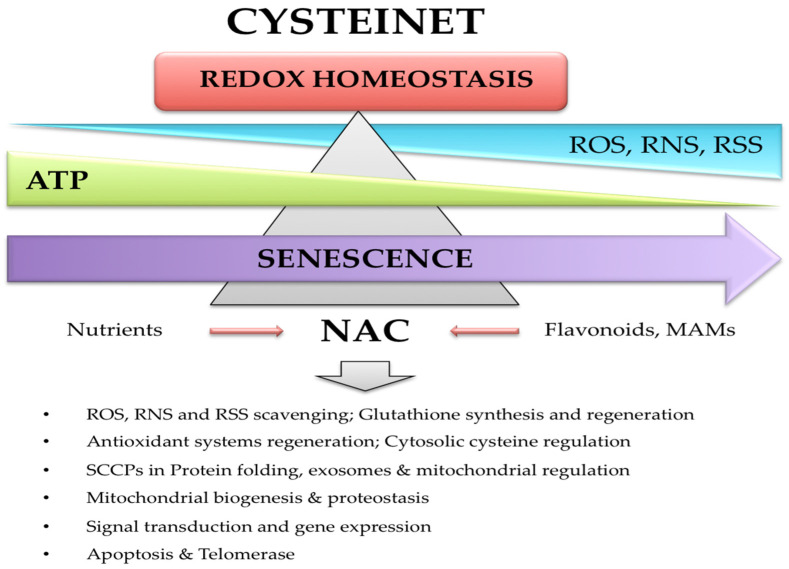
Cysteinet deregulation in aging and Parkinson’s disease. Senescence is associated with a deregulation of reactive oxygen (ROS), nitrogen (RNS), and sulfide (RSS) species. Moreover, the mitochondrial bioenergetic ability is impaired, contributing to glutathione (GSH) decline and cysteinet deregulation. Chronic NAC supplementation may rejuvenate cysteinet and redox homeostasis in Parkinson’s disease.

Maintaining redox regulation at the protein level is challenging due to the numerous metabolic and cell signaling pathways involved [17,20,21,22,23,24,41]. Any compound that can interfere with the redox proteome must be considered a powerful medication, even at low doses. Unlike GSH, NAC has better oral and topical bioavailability, and has been commercially accessible for a long time [17]. We suggested that NAC regulates SCCPs in a comprehensive range of pathways involved in neurodegenerative and psychiatric disorders [17,20,21,22,23,24,41,191,192,193,194,195,196,197,198,199,200,201,202,203]. Moreover, NAC may work synergistically with other supplemental nutrients, such as glycine [204]. Although there is still some controversy about the doses, NAC is a supplement that increases GSH levels contributing to decreasing oxidative damage. Since NAC is a membrane-permeable cysteine precursor, it does not need the alanine–serine–cysteine system to enter the cell, yielding cysteine to regenerate total GSH content and reducing excessive GSSG (Figure 5).

A unique intravenous dose of NAC augmented the blood GSH/GSSG ratio and total GSH in the brain, showing a positive direct correlation between the blood ratio and brain GSH concentrations [205]. However, oral NAC intake is quickly absorbed with a plasma half-life of 2.5 h and no detectable NAC in 10–12 h. Single doses of 600 mg/day and 1200 mg/day result in 16 mM and 35 mM plasma concentrations, respectively [206]. The terminal half-life of reduced NAC is 6.25 h, incorporated into proteins after several hours [206]. There is sufficient clinical evidence of the beneficial action of NAC against oxidative damage in multiple conditions, including oncological and cardiovascular diseases, ophthalmic illnesses, HIV infection, metal toxicity, cerebral ischemic and bleeding disturbances, traumatic brain injury, and neuropsychiatric disorders [207,208,209,210,211,212]. Low NAC supplementation may prevent brain aging and neurodegenerative illnesses, not only via classical antioxidant mechanisms but rejuvenating the cellular redox homeostasis via SCCPs regulation [17,20,21,22,23,24,41].

NAC has shown beneficial effects in neurodegenerative diseases through GSH replenishment and its direct scavenging ability against reactive species [17]. However, its potential significance in modulating the redox proteome and proteostasis is vaguely known. NAC can rejuvenate the specific activities of complexes I, IV, and V in mice presynaptic mitochondria from aged mice. These effects are attributed to the repair of functional cysteines [213,214,215,216]. Moreover, in vivo enzymatic complexes’ specific activities were corrected by chronic NAC supplementation, improving ATP and GSH levels and reducing lipid and protein oxidation markers in pre-synapses [213,214,215,216].

#### 5.3.2. NAC in Cysteinet Regulation

NAC may modulate the redox homeostasis of functional cysteines in numerous proteins, restoring critical cellular pathways to support neuronal survival [24] (Figure 5). For example, NAC activates the Ras-ERK pathway in PC12 cells, preventing neuronal death that lacks trophic factors through nonantioxidant mechanisms. Ras proteins have essential redox-sensitive cysteines suggesting that NAC directly activates Ras via its reducing ability [217,218]. NAC can also protect human neurons against apoptosis induced by Aβamyloid 1–42 [193], starting the p35/Cdk5 pathway and decreasing the phosphorylation/deactivation of MLK3–MKK7–JNK3 signaling (reviewed in [17]). NAC can suppress transcription factors such as NF-κB, inhibiting the next cytokine generation [219]. Moreover, NAC may downregulate the APP gene transcription in neuroblastoma cells by lowering the activity of NF-κB [220]. These NAC effects can be due, at least partially, to sensitive cysteines in those SCCPs [17,24] (Figure 4 and Figure 5).

Redox metabolism regulates transcription factors like NF-κB, AP-1, and the IKK, which include redox-sensitive cysteines [221]. Specifically, NF-κB has Cys38 and Cys62 essential for its function [134,221], IKK possesses Cys179 that intervenes in the kinase activity [222], and the transcription factor AP-1 binds DNA through functional cysteines [223]. Interestingly, NAC can control transcription [214]. NAC can decrease the oxidative damage of NF-κB in clinical sepsis and other oxidative stressing conditions [219,224].

NAC can cross the blood–brain barrier interfering with the main SCCPs in the brain, balancing cysteinet deregulation by replenishing cellular-soluble (H_2_S, cysteine/glutathione) and protein-associated thiols, restoring the mitochondrial bioenergetic power and biogenesis. In addition, NAC increases the level of brain-derived neurotrophic factors sustaining the survival of neurons and stimulating neurogenesis [225,226]. Therefore, NAC must be considered a potent compound, even at low doses, modulating essential protein function from multiple pathways, and consequently, its supplementation has to be accurately specified.

#### 5.3.3. NAC in Protein Misfolding

NAC may rejuvenate age-related protein misfolding and aggregation by stopping cysteines’ oxidative damage. Proteins can suffer conformational modifications when they suffer oxidative injury [227]. In senescence, the balance among protein synthesis, folding, and clearance may shift towards misfolding and aggregation, contributing to neurodegeneration. Cysteine inhibits the assembly and accumulation of amyloidogenic peptides [228], and the beneficial effect of NAC on protein aggregation was confirmed in a mouse model of Huntington’s disease [229]. The changes in the 3-D structure of proteins occur when they accumulate in tissues, mainly in proteins with repetitive amino acids, like polyglutamine in Huntington’s disease [229]. Therefore, NAC can partially fix the aggregation of protein neutralizing misfolding mechanisms in neurodegenerative disorders. Accordingly, cysteine inhibits the fibrillation of Aβ1–40 and Aβ1–42, and their cysteine-induced aggregates were less toxic than those generated by catechin [228].

#### 5.3.4. NAC in Cellular Vesicle Regulation and Signaling

The oxidative control of reactive cysteines on proteins can drive exosomes’ formation and functions. NAC supplementation can rejuvenate vesicular secretion and composition [57]. Thus, a beneficial mechanism of NAC administration in PD may be the replenishment of cysteine thiols to restore the redox regulation of extracellular vesicles. NAC and N-acetylcysteine amide (NACA) can suppress exosome building by scavenging thiol groups, preventing their reaction with cellular thiols. Specifically, NAC may repair exosome secretion, composition, and functions under stressing conditions to physiological levels, controlling oxidative modifications in exosome signaling and aging [230,231].

On the other hand, CSPα is an SCCP that participates in synaptic vesicle endocytosis and synaptic neurotransmission [232]. CSPα oligomerization relies on a cysteine-rich domain used for attachment to synaptic vesicles [232]. The α-synuclein also functions as a chaperone-like protein in combination with CSPα for building the SNARE complex [233]. This α-synuclein process concerns the association with synaptotagmin, which have reactive cysteines in their structure [233,234].

Therefore, thiol-supplying substances may protect against the detrimental disturbance of cellular vesicle formation under oxidative states, supporting the suitability of NAC as a preventive and restorative compound against neurodegeneration in aging and associated disorders via cysteinet rejuvenation.

#### 5.3.5. NAC and Mitochondria

Oxidative damage in synaptic mitochondria is associated with brain senescence [213,214,215,216], likely affecting critical cysteines in mitochondrial proteins. We think the redox equalization of mitochondrial SCCPs may explain many effects of NAC chronic implementation at low doses in the brain [17,24]. Investigations in presynaptic mitochondria from aged mice chronically supplemented with NAC indicated its rejuvenation properties, boosting ATP by activating mitochondrial respiration and oxidative phosphorylation, raising GSH levels, and diminishing lipid and protein oxidations [20,21,22,23,24,41,193]. Adequate NAC levels in the aged brain maintain glutamate uptake via astrocytes and neurons expressing the excitatory amino acid carrier-1. NAC switched the GSH deficit and oxidative damage in the defective mouse of these carriers, indicating that the excitatory amino acid carrier-1 is required for cysteine uptake and GSH synthesis in the brain [235].

#### 5.3.6. NAC and Protein Kinases

NAC may rejuvenate vital pathways against neural death, such as the Ras–ERK (extracellular signal-regulated kinase) pathway, with nonantioxidant effects when trophic factors are absent [17,18,24]. Ras possesses functional cysteines involved in redox-regulated mechanisms [212,213]. In this regard, NAC protects human cortical cerebral neurons against Aβ-amyloid 1-42 [193], inducing p35/Cdk5 action and declining phosphorylation of the MLK3-MKK7-JNK3 signaling pathway. Cdk5 is a cyclin-dependent kinase [236,237,238] involved in neuroprotection, neuronal migration, axonal guidance, and synaptic spine density [239]. Cdk5 dysfunction participates in the pathophysiology of various neurodegenerative conditions, including PD [240]. Cdk5 activation via proteolysis of p35 to p25 via calpain contributes to neuronal toxicity. Indeed, Cys83 and Cys157 s-nitrosylation triggers SNO-Cdk5 building, inducing β-amyloid dendritic spine drop [240]. Also, SNO-Cdk5 is present in AD brains compared to controls, indicating that SNO-Cdk5 may disrupt its activity and thus participating in neuronal death [240].

Mixed lineage kinase 3 (MLK3) is activated via s-nitrosylation at the sensitive Cys688, contributing to its activation and brain ischemia/reperfusion damage [241,242]. In this regard, NAC can inhibit MLK3 activation in early ischemia/reperfusion phases following brain hypoxia [242]. Interestingly, MLK3 can phosphorylate other SCCPs, such as Pin1 [243], increasing their function and translocation to the nucleus [244].

#### 5.3.7. NAC and Telomerase

Increased ROS may affect hTERT localization and activity, which are reverted by low doses of NAC, preventing hTERT translocation from the nucleus to the cytosol and endothelial cell aging [245]. Interestingly, hTERT functionality can suffer post-transcriptional regulation via protein kinases such as c-Abl, PKC, ERK1/2, and Akt [246], some of which are SCCPs. Therefore, they are susceptible to being modified by NAC. Another study indicates that chronic NAC therapy decreases cultured endothelial cell aging by hampering telomere erosion via partial hTERT activation, suggesting that the beneficial effect of NAC depends on the gain of the hTERT function [247]. Also, NAC started the translocation and activity of the cytoplasm to the nucleus hTERT when aging was delayed [247].

#### 5.3.8. NAC-GSH and Nanotechnology

The supplementation of GSH and its precursors, such as cysteine, has some issues because of their instability and toxicity. In this regard, the usefulness of NAC lipophilic products enhanced their tissue biodistribution. Another alternative for improving GSH bioavailability is employing delivery systems such as liposomes, microemulsions, nanoparticles, and microparticles prepared with natural or synthetic polymers, with promising results in humans [248]. There are also investigations using GSH to implement medications into the brain or to control medicine delivery in the intracellular compartment [248].

## 6. Preclinical and Clinical Studies of NAC in PD

Growing proof has established the beneficial effects of NAC in different neurological disorders [17,24]. Concerning the effects of NAC in preclinical PD models, oxidative stressing states and toxic-induced α-synuclein conditions result in c-Abl activation, which is reversed by NAC administration, improving dopaminergic neuronal survival and decreasing motor symptoms [249]. Moreover, NAC can inhibit the drug-induced oxidative injury of peroxiredoxins in dopaminergic neurons, suggesting that sensitive cysteines are involved in the vulnerability of peroxiredoxins to oxidative damage in PD models [250]. The elucidation of which proteins are redox-deregulated in PD will shed light on cysteine-dependent pathways at distinct cellular compartments. Consequently, the work of Monti et al. [251,252] using NAC in PD patients may acquire significant relevance [20,22,196,198]. Patients with idiopathic PD supplemented with NAC therapy showed a significant improvement in DAT binding estimated with DaTscan SPECT imaging associated with decreased symptomatology of the disease [251,252].

However, a short study in mild-to-moderate PD and controls taking extremely high doses of NAC (3000 mg taken orally twice daily) for four weeks showed a boost in cysteine levels and antioxidant criteria but no amelioration in oxidative stress parameters or change in brain GSH levels [253]. More importantly, some participants underwent an increase in PD symptoms ameliorated after discontinuing NAC intake. These contradictory results potentially highlight the different doses (very high doses versus chronic low or moderate ones), periods of NAC administration, and differences in the disease status of selected patients.

Excitatory amino acid transporter 3 (EAAT3)-deficient mice show low levels of neuronal GSH, increased oxidative damage, and death in nigro-striatal dopaminergic neurons during aging [254]. The EAATs dysfunction produces excitotoxicity-associated injury in several neurological conditions [255]. In addition to removing the excitatory amino acid from the synaptic space, EAAT3 also transports cysteine with high affinity [256], supporting the intracellular redox status [257]. Treatment of EAAT3-deficient mice with NAC retrieved the normal phenotype, indicating that EAAT3 function is crucial for cysteine redox homeostasis [254,257].

PD patients supplemented with whey protein showed a significant increase in blood GSH levels and GSH/GSSG ratio [165]. One study in PD patients taking 1000 mg of omega-3 fatty acids from flaxseed oil and vitamin E for 12 weeks showed increased GSH levels, total antioxidant ability, and decreased inflammatory markers [258].

The MPP^+^ inhibits the mitochondrial complex I resulting in ATP deficit and cell death. Zerumbone can raise PARK7, Nrf2, and HO-1 concentrations, enhancing the viability of human neuroblastoma cells treated with MPP^+^ and decreasing ROS content and apoptosis [180].

## 7. Conclusions and Future Perspectives

The central role of oxidative stress in PD was shown a long time ago [13,14,52,259,260,261], including its harmful effects on specific proteins implicated in the pathophysiology of the disease [24,92,109,250]. Likewise, aging impairs oxidative phosphorylation, decreases GSH levels, and increases oxidative species in the mitochondrial dopaminergic system [13,14]. Indeed, low levels of GSH seem to be the first biochemical event identified in the substantia nigra of early PD patients, which may contribute to the damage of susceptible proteins via oxidative modifications [259]. Also, a decreased reduced/oxidized glutathione ratio correlates with the severity of PD, and it is the earliest indicator of dopaminergic neurodegeneration associated with mitochondrial complex I deficiency [260,261].

The accumulation of oxidative damage decreases the antioxidant ability impairing the mitochondrial energetic power in the brain via mechanisms that include protein oxidation and the deregulation of the cysteine proteome, which are the basis for supplying GSH precursors in PD. Any therapy contributing to the redox proteome rejuvenation can potentially modulate highly regulated biological processes but can also have harmful secondary effects [24]. In this regard, chronic low doses of NAC seem to be a secure and natural cysteine precursor modulating GSH levels, redox homeostasis, and adequate SCCPs function, including GSH, which is a sensitive cysteine-containing tripeptide that relies on the cysteine/cystine status and cellular redox conditions [17,24]. An important issue that has not been definitively resolved is the contribution of astrocytes to the maintenance of neuronal GSH status. It is a very complex subject in which in vitro and in vivo studies present some contradictions that have been recently reviewed in depth [262].

NAC is the best and simplest known compound, broadly used in clinical practice that can cross the blood–brain barrier to rejuvenate redox homeostasis and SCCP function in the brain, decreasing the detrimental effects of aging on neuronal survival [226]. Indeed, clinical studies have demonstrated the rejuvenating impact of NAC on the dopamine system and symptomatology in PD [251,252]. NAC can directly modify the in vitro and in vivo activity of transcription factors such as NFκB, decreasing the following cytokine production [209], and downregulating the APP gene transcription [220]. These effects are likely mediated through the proposed cysteinet regulation [17,22,24].

Albumin is the main SCCP in the blood, playing a central function in redox homeostasis via the functional Cys34, serving as a predictive biomarker of oxidative deterioration [263,264] and contributing to cysteinet regulation in human aging and neurodegenerative conditions. Redox Cys34 status in albumin is an index that may predict the advancement of oxidative injury in chronic degenerative disorders [263,264]. Oxidized albumin is about 35% in physiological conditions, rising to 70% under some oxidative insults [265]. The Cys34 in albumin interacts with the cysteine/cystine balance without any enzymatic involvement [265]. Similarly, the function of many SCCPs depends on the cysteine/cystine status in the blood, CSF, and brain, controlling aging and age-associated neurodegenerative development.

Although the effects of NAC on oxidative stress and redox homeostasis are not fully understood, there are some crucial resolved points. First, NAC has antioxidant properties and provides cysteine for GSH synthesis. Second, NAC can modulate the redox metabolism working on functional cysteines of many SCCPs. Third, high doses of NAC can be detrimental, increasing adverse effects. Fourth, diets rich in vitamins and some vegetables could potentiate NAC levels and effects. Fifth, NAC can cross the blood–brain barrier and directly interact with vital SCCPs in the brain. Sixth, NAC can restore redox dysfunction by replenishing mitochondrial-soluble and protein-linked thiols, reducing oxidative damage in brain aging.

The involvement of SCCPs in PD pathophysiology is constantly growing. Oxidative injury and disulfide bridges in tau and other SCCPs are crucial pathogenic factors [266], and APOE4 and tau implication in PD dementia has lately been identified [267]. Hence, targeting the cysteine redox proteome is a potential preventive strategy against PD initiation and evolution [268].

Therefore, we suggest that the cellular rejuvenation of the cysteine redox proteome using GSH precursors is an underestimated multitarget therapeutic approach against neurodegeneration in PD, supporting the performance of long-term clinical studies:

- large-scale clinical trials;
- including PD patients, healthy people, and age-matched patients with comorbidities (e.g., diabetes, hypertension, obesity, and cardiovascular disorders);
- supplementing the diet with cysteine-rich food or low doses of NAC (between 1800 and 3000 mg/week);
- monitoring GSH and albumin redox status.

## Data Availability

Not applicable.

## Abbreviations

PD, Parkinson’s disease; NAC, N-acetyl-cysteine; GSH, glutathione; ROS, reactive oxygen species; RNS, reactive nitrogen species; RSS, reactive sulfur species; SCCPs, sensitive cysteine-containing proteins; PTMPs, post-translational modification of proteins; -SH, thiol; GSSG, glutathione disulfide; PRC2, polycomb repressive complex 2; Keap1, Kelch-like ECH-associated protein 1; Nrf2, nuclear factor erythroid 2-related factor 2; γ-GS, γ-glutamate-cysteine synthetase; GR, glutathione reductase; GPX, glutathione peroxidase; Ero1, endoplasmic oxidoreductin 1; PDI, protein disulfide isomerase; MAPK, mitogen-activated protein kinase; ATM, ataxia-telangiectasia mutated; BMP7, bone morphogenetic protein 7; c-Abl, cellular-abelson tyrosine kinase; CSPα, cysteine string protein α; DJ-1, protein deglycase DJ-1; ETC, electron transport chain; GCase, glucocerebrosidase; GLI3, GLI family zinc finger 3; GSR, glutathione-disulfide reductase; IP3, inositol-1,3,5-triphosphate; IP3R, IP3 receptor; MSH2, MutS homolog 2; NF-κB, nuclear factor kappa light chain enhancer of activated B cells; OCT4, octamer-binding transcription factor 4; Parkin, E3 ubiquitin ligase; PRX, peroxiredoxin; RFC, replication factor C; TOP3B, DNA Topoisomerase III beta; TPP1, tripeptidyl-peptidase 1; TXN, thioredoxin; RYRs, ryanodine receptors; DNAJC, dnaJ homolog subfamily C member; HSP, shock protein; SNARE, soluble NSF attachment protein receptor; LRRK2, leucine-rich repeat kinase 2; PARK8, autosomal dominant Parkinson disease-8; GTPase, guanosine triphosphatase; Rab10, Ras superfamily of small G protein 10; DAT, dopamine transporter; PTEN, phosphatase and tensin homolog; SUR, sulfonylurea receptor; K-ATP channel, ATP-sensitive potassium channel; MAP2, microtubule-associated protein 2; Mfn1, mitofusin-1; Mfn2, mitofusin-2; Opa1, optic atrophy type 1; NSCs, neural stem cells; AP-1, activator protein 1; HMTase, histone methyltransferase; DSBs, double-strand breaks; MPP^+^, 1-methyl-4-phenylpyridinium; DDR, DNA damage response; TGF-β, transforming growth factor β; hTERT, human telomerase reverse transcriptase; NO, nitric oxide; IL-1β, interleukin-1 beta; TNF-α, tumor necrosis factor-alfa; MAMs, Michael acceptor molecules; IKK, factor-kappa B kinase; PKC, protein kinase C; NACA, N-acetylcysteine amide; SNO-Cdk5, s-nitrosylation of cyclin-dependent kinase 5; MLK3, mixed lineage kinase 3; Pin 1, peptidyl-prolyl cis-trans isomerase NIMA-interacting 1; ERKs, extracellular signal-regulated kinases; EAAT3, excitatory amino acid transporter 3; APOE4, apolipoprotein E4.
